# Dual-species transcriptional profiling during systemic candidiasis reveals organ-specific host-pathogen interactions

**DOI:** 10.1038/srep36055

**Published:** 2016-11-03

**Authors:** Betty Hebecker, Sebastian Vlaic, Theresia Conrad, Michael Bauer, Sascha Brunke, Mario Kapitan, Jörg Linde, Bernhard Hube, Ilse D. Jacobsen

**Affiliations:** 1 Research Group Microbial Immunology, Leibniz Institute for Natural Product Research and Infection Biology (Hans Knöll Institute), Jena, Germany; 2 Center for Sepsis Control and Care, Jena University Hospital, Jena, Germany; 3 Department of General, Visceral and Vascular Surgery, Experimental Transplantation Surgery, Jena University Hospital, Jena, Germany; 4 Research Group Systems Biology/Bioinformatics, Leibniz Institute for Natural Product Research and Infection Biology (Hans Knöll Institute), Jena, Germany; 5 Department of Bioinformatics, Friedrich-Schiller-University Jena, Germany; 6 Department of Anaesthesiology and Intensive Care Therapy, Jena University Hospital, Jena, Germany; 7 Department of Microbial Pathogenicity Mechanisms, Leibniz Institute for Natural Product Research and Infection Biology (Hans Knöll Institute), Jena, Germany; 8 Friedrich-Schiller-University, Jena, Germany

## Abstract

*Candida albicans* is a common cause of life-threatening fungal bloodstream infections. In the murine model of systemic candidiasis, the kidney is the primary target organ while the fungal load declines over time in liver and spleen. To better understand these organ-specific differences in host-pathogen interaction, we performed gene expression profiling of murine kidney, liver and spleen and determined the fungal transcriptome in liver and kidney. We observed a delayed transcriptional immune response accompanied by late induction of fungal stress response genes in the kidneys. In contrast, early upregulation of the proinflammatory response in the liver was associated with a fungal transcriptome resembling response to phagocytosis, suggesting that phagocytes contribute significantly to fungal control in the liver. Notably, *C. albicans* hypha-associated genes were upregulated in the absence of visible filamentation in the liver, indicating an uncoupling of gene expression and morphology and a morphology-independent effect by hypha-associated genes in this organ. Consistently, integration of host and pathogen transcriptional data in an inter-species gene regulatory network indicated connections of *C. albicans* cell wall remodelling and metabolism to the organ-specific immune responses.

*Candida albicans* is the most common cause of life-threatening fungal bloodstream and disseminated infections. The crude mortality of disseminated infections caused by *Candida* is >50%, higher than for bacterial blood stream infection^1^. The murine model of hematogenously disseminated candidiasis is most commonly used to study systemic candidiasis and to evaluate efficacy of antifungal therapy. Following intravenous infection, *C. albicans* initially infects almost all organs; however, while the fungal load increases over time in kidneys, it declines in liver and spleen^2^,^3^. Moreover, *C. albicans* is able to form filaments – a hallmark of pathogenicity - in the kidney within two hours after intravenous infection, whereas no hypha formation is observed in liver and spleen^4^. Thus, the kidney appears to be the prime target organ for disseminated candidiasis in mice. In contrast, disseminated candidiasis in humans affects kidneys but also commonly leads to infection of liver and spleen^5^.

The relative susceptibility of the kidneys in murine candidiasis has been linked to the specific immunological setup of this organ: Neutrophils and macrophages are present in higher numbers in liver and spleen than in the kidneys of naive animals^2^. In addition, in comparison to spleen and liver, leukocytes are recruited later to *C. albicans*-infected kidneys^2^. Early accumulation of neutrophils has a protective effect and mononuclear phagocytes can directly kill *C. albicans in vitro* and *in vivo*^2^,^6^,^7^. As kidney-resident macrophages and inflammatory monocytes are important for fungal clearance in the kidney, the delay in the early phagocytic response in the kidney seems to be critical for progression of infection^6^,^7^. However, at later stages of infection continued neutrophil recruitment induced by locally produced cytokines and chemokines in the kidney directly contributes to immunopathology^3^. Thus, the immune response, fungal clearance and pathological consequences vary significantly in different organs during systemic *C. albicans* infection in the murine model.

*C. albicans* expresses a variety of virulence factors that contribute to pathogenesis, including the morphological transition between yeast and hypha, the expression of hypha-associated virulence factors and metabolic flexibility^6^,^8^,^9^. In addition to morphogenesis, the *C. albicans* cell wall structure is strongly influenced by environmental factors, such as available carbon sources or environmental stresses, leading to rapid cell wall remodelling processes *in vitro* and *in vivo* that affect interaction with immune cells^10^,^11^,^12^. Furthermore, *C. albicans* rapidly adapts to the changing environments encountered during infection by changes in gene expression, translation, and post-translational modifications^13^,^14^.

Thus, both the host and the fungus respond rapidly to the changing conditions during systemic candidiasis. Such changes can be monitored on the transcriptional level by *in vivo* gene expression profiling to estimate the functional adjustments during invasive growth of *C. albicans*. Recently, Xu *et al*. analysed 479 *C. albicans* and 46 mouse genes from kidney samples using the NanoString technology^15^. Although less than 2% of the *Candida* genome was covered, a clear induction of *C. albicans* hypha-associated as well as zinc and iron responsive genes was observed. Similarly, RNASeq of *C. albicans* cells infecting the mouse kidney revealed regulation of iron metabolism, hypha formation, and metabolic adaptation during infection^16^.

Even though these studies significantly contributed to our understanding of disseminated candidiasis, they focused on the kidney as the main target organ. However, given the different immune responses in different organs, it is likely that organ-specific host-pathogen interactions occur and contribute to the different course of infection in murine organs. Understanding these differences may be crucial in understanding pathogenicity of life-threatening systemic *C. albicans* infections. Therefore, we performed a time-course transcriptional analysis of liver, spleen, and kidney samples from mice infected intravenously with *C. albicans*. Our results demonstrate not only a delayed immune response in the kidney compared to liver and spleen, but also qualitative differences in the responses of these organs. Microarray analyses of *C. albicans* cells likewise showed organ-specific adaptation processes. This includes an increased expression of hypha-associated genes in the liver in the absence of visible filamentation, suggesting that gene expression and morphogenesis is uncoupled under these specific conditions *in vivo*.

## Results and Discussion

### Murine spleen, liver, and kidney show different kinetics of transcriptional responses to systemic candidiasis

Transcriptional profiling is a powerful tool to elucidate host-pathogen interactions during infection^17^,^18^ that has been successfully applied to the murine model of disseminated candidiasis^15^,^16^,^19^,^20^,^21^. However, previous studies focused on the kidney as the main target organ. Given the differing progression of infection in kidney, liver, and spleen in mice and the frequent involvement of liver and spleen in human disseminated candidiasis, we performed a transcriptional profiling of these three organs over the time course of infection in mice.

**Figure 1:**
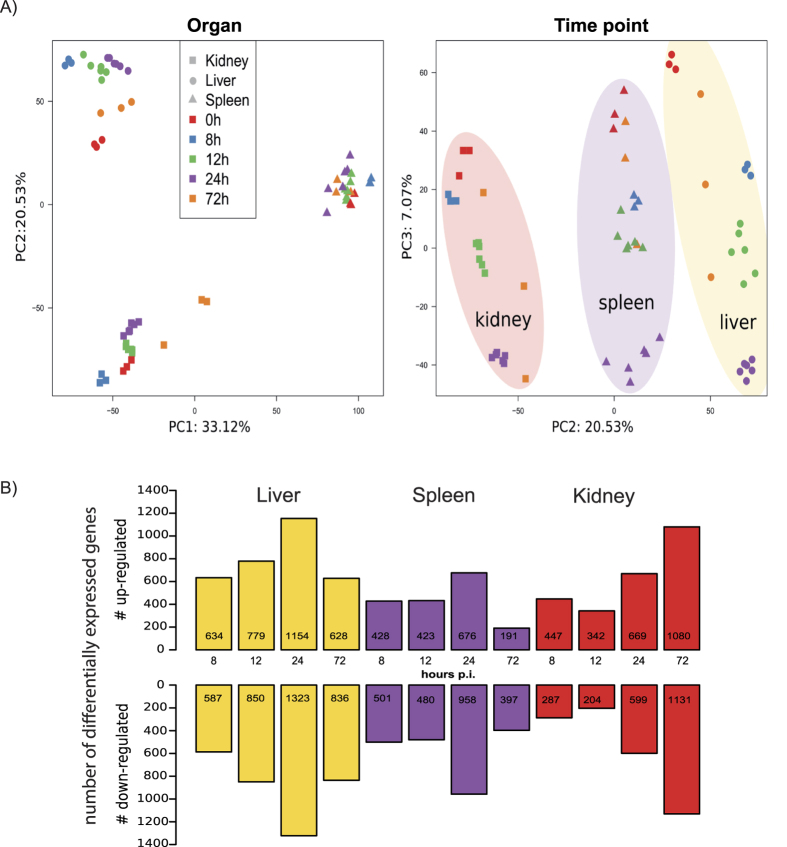
Principle component (PC) analysis of mouse expression data (A) and numbers of differentially expressed genes (DEGs) (B). (**A**) Left: Approximately 54% of the variation between data sets was captured by the first two PCs and coincided with the organ the sample was obtained from. Right: The third PC captured about 7% of the variance in the data and correlated with the temporal effects in the data set. (**B**) Numbers of genes differentially regulated (p < 0.05) in kidneys, liver and spleen during *C. albicans* infection as compared to the PBS control.

**Figure 2:**
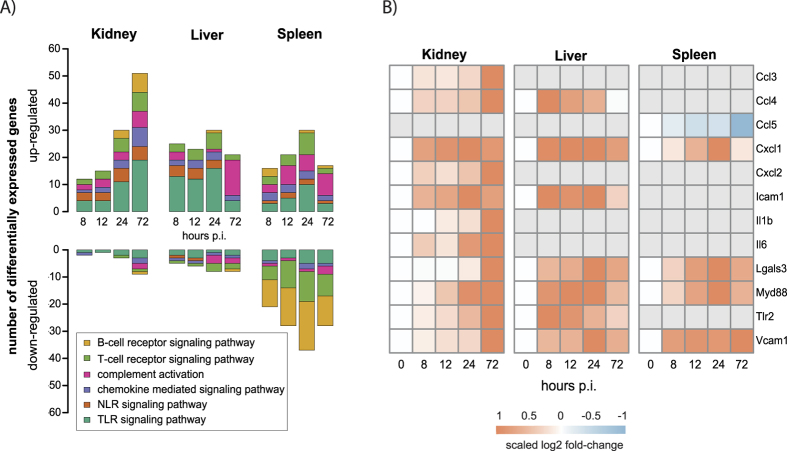
Gene expression changes in immune response pathways after systemic *C. albicans* infection. (**A**) Number of differentially regulated genes in distinct immune pathways. The org.Mm.eg.db package for R was used to identify differentially expressed genes associated with the pathways “TLR signaling pathway” (GO:0002224), “NLR signaling pathway” (GO:0035872), “chemokine mediated signaling pathway” (GO:0070098), “complement activation” (GO:0006956), “T-cell receptor signaling pathway” (GO:0050852) and “B-cell receptor signaling pathway” (GO:0050853) including associated child-terms, respectively. (**B**) Gene expression of selected differentially expressed immune genes. For each organ, log2 fold-changes were gene-wise maximum scaled between −1 and 1 (scaled log2 FC). Measurements of probes mapping to the same gene were mean averaged.

Principal component analysis (PCA) of the murine transcriptional data sets showed that 54% of the variance (PC1 and PC2) among all transcripts in the data sets could be attributed to organ-specific expression differences (Fig. 1A). Temporal changes also contributed to variance (7%) and biological replicates from individual organs clustered according to time point (Fig. 1A). PCA reflects general expression trends in the data which are due to alterations in the expression of the majority of genes represented on the microarray. When specifically analyzing differentially expressed genes (DEGs), organ-specific kinetics of transcriptional changes became evident: Most of the changes in gene expression in liver and spleen were observed already 24 h p.i., whereas in kidney the number of DEGs continuously increased over time (Fig. 1B). We thus performed cluster analysis to categorize the genes according to organ expression kinetics and analysed these clusters for enriched GO terms. All organs responded to infection by regulation of genes associated with immune processes and stress response (Suppl. Fig. 1A, Suppl. data 1 and 2) and, more specifically, upregulation of genes associated with the response to interferon β and cytokine biosynthesis (represented by cluster 5, Suppl. Fig. 1B). However, the expression kinetics differed between organs: As shown in Fig. 2A, several genes in TLR and NLR pathways were upregulated from 8 h to 24 h p.i. in the liver, while in the kidney sizable induction of these pathways was detected only after 24 h and further increased towards 72 h p.i. At 72 h p.i., complement activation was significantly induced in the liver. In the spleen, especially genes in T- and B-cell receptor signalling pathways were enriched; however, the involved genes were mainly downregulated compared to the control. The subset of genes displayed in Fig. 2B highlights the differences in the expression kinetics of genes associated with inflammation: Expression of most of these genes in the liver increased strongly early after infection with a tendency to return to control sample expression levels at later time points, whereas in kidney samples the largest changes were detected 72 h p.i.

Thus, while all organs responded to infection by downregulation of genes associated with organ-specific functions and upregulation of immune processes, the kinetics and type of immune responses differed.

### Organ-specific transcriptional changes in systemic candidiasis reflect activation of local immune responses and organ damage

As discussed above, the transcriptional changes in the kidney were characterised by late upregulation of proinflammatory pathways, consistent with the reported delayed recruitment of immune cells in this organ^2^. Notable exceptions were the chemokine CXCL1 (also known as keratinocyte cell–derived chemokine [KC]) and the intercellular adhesion molecule ICAM-1, which is important for leukocyte transmigration. These genes were highly upregulated after 8 h in the kidney (Fig. 2B). As we analysed the transcriptome of whole organs, the precise cellular source of early immune transcripts is not clear. It has, however, been shown that CXCL1 is mainly produced by resident tissue macrophages via TLR4 signalling through MyD88^22^. In comparison to liver and spleen, the number of resident immune cells in the kidney is low^2^ and even though transcript abundance of CXCL1 increased in the kidney 8 h p.i., protein levels are known to increase more steeply only after 24 h^3^. This suggests that the potential of resident renal macrophages to produce this chemokine is not sufficient to drive early neutrophil recruitment at high levels. The progressive upregulation of proinflammatory pathways in the kidney over the course of infection can be explained partially by the increasing or persistent fungal burden which leads to ongoing immune stimulation. Fungal growth is furthermore associated with progressive renal damage^3^,^23^, reflected in our data set by (i) the upregulation of genes involved in wound healing and (ii) the downregulation of genes in “renal system processes”, “transport processes”, and “ion homeostasis” observed at later time points (Suppl. Fig. 1).

Organ-specific immune responses included the induction of the acute phase response in the liver (Suppl. Fig. 1A) and the TGF-β pathway and genes associated with lymphocyte activation and leukocyte proliferation in the kidney (Suppl. Fig. 1A). While the latter were upregulated at late time points in the kidney, their expression was continuously decreasing until 24 h in the spleen, with a tendency to return to basal levels towards 72 h p.i. As a secondary lymphoid organ, main functions of the spleen are associated with leukocyte activation and proliferation. Therefore, transiently reduced expression of these genes could be interpreted as reduced expression of genes with organ-specific functions. The transient nature of these alterations might thus reflect the successful control of fungal growth in the spleen. Downregulation was also observed in the liver for genes involved in liver function (“monocarboxylic acid metabolism” and “lipid homeostasis”, cluster 9, Suppl. Fig. 1). While these genes also showed a trend to return to basal levels at 72 h p.i., it was not as pronounced as for the spleen, indicating that the transcriptional response of this organ was continuing even though infection is controlled to a similar degree in liver and spleen. Furthermore, although the kinetics of fungal burden and immune cell recruitment in liver and spleen are similar during systemic candidiasis, the transcriptional immune response of these organs differed significantly: Components of the innate immune response, such as TLR and NLR signalling pathways, were induced to a higher extend in the liver. The lack of strong induction of genes associated with proinflammatory responses is consistent with the comparatively low and transient production of proinflammatory cytokines in the spleen during systemic candidiasis^3^. We find it noteworthy, however, that systemic responses were clearly induced in the liver, e.g. the acute phase response, and that genes involved in complement activation were upregulated especially after 72 h p.i. This might indicate the role of the liver for the systemic immune response during ongoing candidiasis independent of local pathogen control, and might explain why expression of genes involved in metabolic liver function (cluster 9, Suppl. Fig. 1) did not return to steady-state levels by 72 h p.

In summary, all organs showed downregulation of genes associated with organ function, likely as a result of organ impairment and/or as consequence of upregulation of genes dedicated to immune responses. Consistent with the development of fungal burden, these changes were transient in spleen and liver but increased over time in the kidney.

### Transcriptional profiles of *C. albicans* indicate organ specific fungal adaptation in kidney and liver

One of the largest challenges for *in vivo* fungal transcription analysis is the relative abundance of host transcripts hampering the recovery of sufficient amounts of fungal RNA^17^. We used an enrichment protocol based on sequential lysis of host and fungal cells on ice. Determination of genome wide transcription levels by microarrays and of selected genes (*TSP1*, *HSP90*, *HSP104*) by quantitative RT-PCR (q RT-PCR) revealed approximately 10% variation between RNA isolated from spiked murine organs using our enrichment protocol and RNA isolated from the spiking culture by standard methods^24^. We were able to isolate sufficient amounts of high-quality fungal RNA to perform microarray analysis from *C. albicans* cells retrieved from the liver 8 h p.i., the kidneys 12 h p.i., and the kidneys 24 h p.i., enabling us to detect 6569 genes in the arrays. The amounts recovered at other time points were sufficient only for q RT-PCR.

The lower amounts of fungal RNA obtained from the kidney at 8 h p.i. or the liver at later time points are possibly due to lower amounts of total fungal biomass and RNA at these time points in the organs. We furthermore detected no cross-hybridization of uninfected kidney samples with the *C. albicans* microarrays, indicating that the detected transcripts originate from *C. albicans*. The microarray data were verified by qPCR analyses of selected genes (Suppl. Table 1).

**Figure 3:**
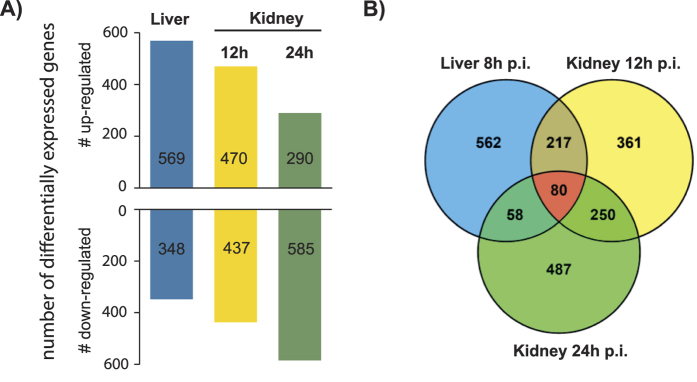
*C. albicans* genes differentially expressed during systemic candidiasis in mice. (**A**) Number of differentially expressed *C. albicans* genes in liver and kidney samples 8 h, 12 h and 24 h p.i. relative to common reference (log phase SC5314 grown in YPD at 37 °C). (**B**) Venn diagram representing the overlap of *C. albicans* DEGs in liver 8 h, kidney 12 h and kidney 24 h p.i. Within all three samples, 80 genes were differentially expressed in all samples, whereas most of the genes were differentially regulated at specific time points or organs.

**Figure 4:**
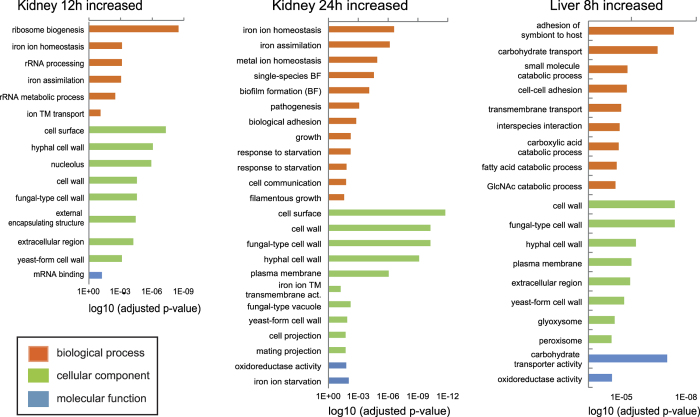
GO terms enriched in positively regulated differentially expressed genes of *C. albicans* during systemic candidiasis. The most significantly regulated ontologies were determined by the adjusted p-value (BP - biological process (orange); CC - cellular component (green), MF - molecular function (blue)). A full list of enriched GO terms is provided in Suppl. data 4.

Compared to YPD culture (control), 917 *C. albicans* genes were differentially expressed in liver, 908 in kidney 12 h p.i., and 875 in kidney 24 h p.i., with 80 genes differentially expressed in all organs (Fig. 3, Suppl. data 3). These included infection-associated, highly upregulated individual genes, such as *HWP1*, *ECE1, ALS1, ALS3, HYR1,* and *SOD5* (Table 1). Furthermore, GO categories related to cell wall and cell surface were significantly enriched in positively regulated genes of all samples (Fig. 4, Suppl. data 4), indicating remodelling of the cell wall and reflecting the upregulation of hypha-associated genes involved in adhesion such as *ALS1*, *ALS3*, *DIF1,* and *HWP1* (Table 1).

While this demonstrates common regulation of a set of virulence-associated genes, most DEGs displayed organ- or time point-specific regulation (Fig. 3B). Some of the organ-specific differences might be due to the differences in the time points analysed (liver 8 h p.i. vs. kidney 12 h and 24 h p.i.), which is a limitation of this study; however, the higher similarity among gene expression profiles at two time points in the kidney compared to the liver (Suppl. Fig. 2A) still suggests organ-specific adaptations.

### The transcriptional response of *C. albicans* during growth in the kidney is characterized by iron acquisition and metabolic adaptation

To elucidate which processes were affected by the DEGs, we performed GO term enrichment analysis and gene set enrichment analysis (GSEA) using published lists of *in vivo* and *in vitro* regulated *C. albicans* genes. Specific expression patterns deduced from GO term enrichment analysis included upregulation of “iron assimilation” in kidney samples at both time points, supported by GSEA results matching gene regulation in the kidney with iron homeostasis^25^ and consistent with two recently published *in vivo* transcriptional profiles of *C. albicans* cells infecting the mouse kidney^15^,^16^. This correlates well with local iron sequestration shown in kidney lesions^26^ and the upregulation of lactoferrin and haptoglobin by the host (Suppl. data 1), supporting the concept of nutritional immunity as a host defence mechanism^27^.

During early kidney infection, according to GO terms *C. albicans* furthermore upregulated translational processes (e.g. “ribosome biogenesis”, Fig. 4), which was supported by GSEA (ribosomal proteins in a set of putative targets of the transcription factor Fhl1^28^). After 24 h in the kidney, the transcriptional profile of *C. albicans* indicated a response to starvation (Fig. 4), possibly as a result of rapid growth and hypha formation. In contrast, metabolic genes differentially expressed in the liver indicated catabolic processes and carbohydrate transport (Fig. 4). Some of the organ-specific metabolic adaptations of *C. albicans* could be a response to organ-specific nutrient supply. For example, while the glycerol biosynthetic gene *RHR2* showed decreased expression in kidney, possibly reflecting the presence of the renal osmoprotectant glycero-phosphocholine^15^, *RHR2* expression was increased in the liver (Suppl. data 3). Indeed, it has been shown that the ability of *C. albicans* to utilize external glycero-phosphocholine is important for virulence in murine systemic candidiasis^29^ and that *RHR2* is essential for proliferation of *C. albicans* in the liver of intraperitoneally infected mice^30^.

### Inference modelling identified three inter-species pathogen-host gene-regulatory networks linking fungal metabolism with the host immune response

Metabolic adaptation of *C. albicans* might also be influenced by immune reactions, especially restriction of nutrient availability upon phagocytosis^13^,^31^. To determine which fungal and host activities may directly influence each other, we inferred an inter-species pathogen-host gene-regulatory network^32^ using the ExTILAR algorithm^33^. This method integrates transcriptional data (DEGs of both species clustered by k-means; Suppl. Fig. 2B,C) with existing knowledge on transcription factor-dependent regulation of the genes to infer a regulatory network between DEGs of host (Suppl. data 5) and pathogen (Suppl. data 6). The resulting network showed three disconnected sub-networks each composed of mouse and *Candida* clusters.

**Figure 5:**
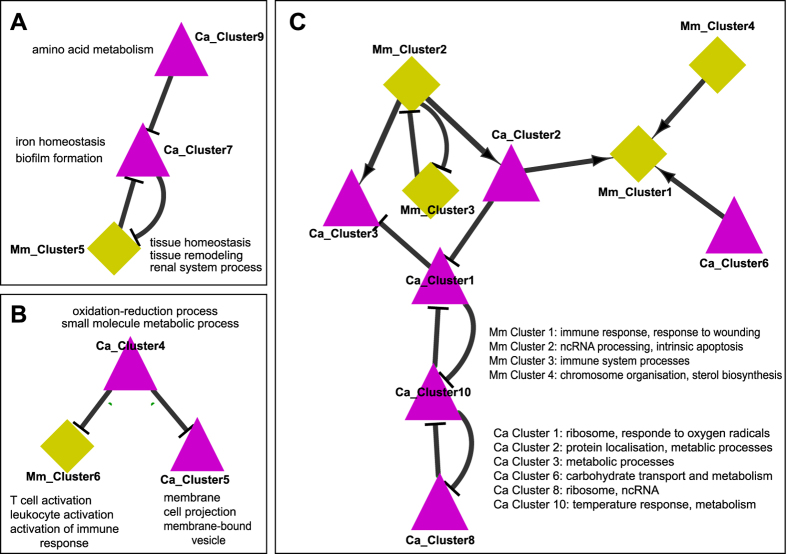
Inferred inter-species pathogen-host gene-regulatory networks. Nodes represent clusters of co-regulated genes for *M. musculus* (diamond) and *C. albicans* (triangle). Edges between two clusters represent directed regulatory interactions that are either positive (arrow) or negative (blunt end). A full list of genes and enriched GO terms of the clusters is provided in Suppl. Data 5 (mouse) and 6 *(C. albicans*).

The first network (Fig. 5A) was composed of a murine cluster containing downregulated genes involved in tissue homeostasis, remodelling and renal function, and thereby reflecting parts of the renal response to infection. This host cluster was both regulated by and regulating a cluster of upregulated fungal genes affecting iron homeostasis and biofilm formation (as observed in the kidney). This *C. albicans* cluster in turn was connected to the second *C. albicans* cluster, comprising downregulated genes involved in amino acid metabolism, similar to the observed changes in the liver. Thus, this sub-network suggests a link between host tissue function on the one side and fungal iron homeostasis and amino acid metabolism on the other, and reflects the organ-specific fungal transcription. Amino acid biosynthesis is affected by iron starvation in *S. cerevisiae* in a complex feedback loop involving transcription factors and biosynthesis gene containing iron-sulfur clusters^34^. Cysteine is important for the formation of iron-sulfur clusters and the *C. albicans* key iron regulator Sfu1 contains a cysteine-rich central domain that is typical for GATA-type transcription factors^35^. Cysteine might thus possibly link Sfu1 and amino acid biosynthesis *via* iron-sulfur clusters; this is however highly speculative. Alternatively, tissue damage caused by invasive fungal growth and the subsequent host response might significantly affect nutrient availability for and metabolism of the fungus.

In the second network (Fig. 5B) the central *C. albicans* cluster contained genes related to metabolic processes that were upregulated in the liver but downregulated in the kidney. This cluster was predicted to negatively affect the expression of genes in both, another fungal and a host gene cluster. In the host cluster, genes were associated with immune system processes and included genes such as IL6, CXCL10 and Interferon- and TNF-receptors, which were upregulated only in the kidney. The second *C. albicans* cluster also showed organ-specific regulation, with increased expression in the kidney and decreased expression in the liver. This cluster was significantly enriched for terms associated with the cell surface and included genes like *CDC12*, *CHS3*, and *MYO2*, that influence filamentation and cell wall composition and thereby could affect interaction with the immune system^36^. Moreover, binding sites for the transcription factor *BCR1*, which regulates biofilm formation and cell-surface associated genes^37^, was over-represented in the promoter region of genes in this cluster. Biofilm-associated genes were also found to be enriched in the kidney by GO term analysis (Fig. 4). Taken together, this sub-network indicates an organ-specific regulatory relationship between host immune responses, fungal metabolism and *C. albicans* morphology.

Similarly, the *C. albicans* clusters within the third network (Fig. 5C) also contained genes with organ-specific transcription patterns and functions for metabolic adaptation, while two of the four connected murine clusters (#1 and #3) were enriched in genes with functions for the immune response and organ-specific transcription.

Thus, all deduced networks support an organ-specific interplay between fungal metabolism and immune processes during infection. Immune reactions likely affect the availability of nutrients, e.g. upon phagocytosis and by iron sequestration^13^,^27^ but metabolic adaptations of *C. albicans* in turn can affect the interaction of the fungus with the immune system^38^.

### Expression of hypha-associated genes in the absence of filamentation in the liver

This link between fungal metabolism and interaction with immune cells is likely to be mediated by changes of the fungal surface and cell wall^38^. Genes involved in biofilm formation were enriched at later time points in the kidney, consistent with observations by others^15^,^16^. GSEA furthermore identified a significant overlap of the *in vivo* transcriptome 24 h p.i. with genes differentially regulated in *C. albicans ccr4*Δ/Δ and *sit4*Δ/Δ^39^,^40^, both involved in cell wall maintenance and filamentation. The cell wall composition of *C. albicans* is also linked to morphogenesis, a central virulence attribute of *C. albicans*, and filamentation occurs early upon systemic infection of the murine kidney^3^. Not surprisingly, filamentation-associated genes were among the most strongly upregulated *Candida* genes in the kidney (Tables 1 and 2). In the liver, however, filamentation does not occur after intravenous infection^2^,^41^ (and own unpublished data). Thus, we were surprised to observe induction of hypha-associated genes (HAGs) and core filamentation response genes^42^ in the liver (Tables 1 and 2). To confirm the results obtained by microarrays, we performed qPCR on our RNA samples to analyse the expression levels of the six genes of the core filamentation response^42^. With the exception of *DCK1*, all core filamentation response genes showed a moderate to strong induction in both organs at all time-points under investigation and, with the exception of *RBT1*, similar expression levels in all samples (Table 2; Suppl. Fig. 3).

### Expression of hypha-associated genes by *C. albicans* yeast cells in the liver could indicate cell wall remodelling

In addition to the core filamentation response genes, several genes encoding cell wall remodelling factors were specifically induced in *C. albicans* in the liver, e.g. *CRH11* (cell wall transglycosylase), *MP65* (cell surface mannoprotein), *PHR1, BGL2* and *PGA4* (glucanosyltransferases), and the chitinase gene *CHT1*.

It is known that the *C. albicans* cell wall is highly dynamic. *In vitro*, the exposure of *C. albicans* to different stresses that are encountered following phagocytosis can lead to rapid architectural changes and structural realignments of the cell wall^43^. Changes observed *in vivo* include altered exposure of chitin and β-glucan^11^,^12^. Both morphology and cell wall composition can influence the interaction with immune cells. For example, murine macrophages phagocytose *C. albicans* yeast cells more efficiently than hyphae^44^ and preferentially ingest O- and N-linked mannan-deficient mutants^14^. Cell wall glycosylation also affects phagosome maturation in macrophages^45^. We thus hypothesized that the induction of HAGs in yeast cells, as observed in the liver, could influence the interaction of the fungus with macrophages. To test this hypothesis, we inoculated log-phase yeast cells grown in YPD (0 min) into RPMI at 37 °C. In this condition, increased expression of *ALS3*, *ECE1*, *HWP1,* and *IHD1* was detected by qPCR as early as 15 min after induction (data not shown), even though first germ tube formation only became visible after 45 min (Suppl. Fig. 4A). These *C. albicans* yeasts were then used in a phagocytosis assay with human monocyte-derived macrophages. We observed a significant increase in the phagocytosis rate of MDMs challenged with RPMI-induced yeast cells (Suppl. Fig. 4B,C) and alterations in the release of cytokines by macrophages stimulated with thimerosal-killed cells (Suppl. Fig. 4D). While this suggests that the induction of the filamentation program is indeed associated with changes that influence the interaction with immune cells, this hypothesis requires further investigation.

It has, however, been well established that both phagocytosis and cytokine production depend on the interaction of the fungal surface and components of the cell wall with host cell pattern recognition receptors. As the cell wall of *C. albicans* responds to numerous environmental cues^45^, it is plausible that the yeast cells found in the liver differ from both, cells grown *in vitro* under yeast-favouring conditions and the yeasts grown under hypha-inducing conditions in our *in vitro* experiment. Morphology-independent expression of HAGs has also been observed during growth in the intestinal tract^46^, suggesting that hypha-like modification of the yeast surface can occur in specific host niches, and GSEA revealed a significant overlap of DEGs in the liver with genes upregulated in the caecum^46^. It is however difficult to predict to which extent the interaction with immune cells in the liver might be influenced by the observed changes of the transcriptome. Given that HAGs were upregulated in the liver 8 h p.i., after prolonged exposure to a likely hypha-inducing environment, it nonetheless appears striking that visible hypha were absent in this organ – especially as they readily form in the kidney and *in vitro* within this time span. Possibly, phagocytosis of *C. albicans* yeasts in the liver exposes the fungus to an environment that prevents hyphal growth.

### *C. albicans* likely faces phagocytosis early after infection of the liver

According to GSEA, the greatest overlap of analysed transcriptomes was observed between *C. albicans* DEGs specific for liver and the transcriptional response to phagocytosis by bone marrow-derived macrophages (Suppl. data 7)^47^. The upregulation of catabolic and transport processes by *C. albicans* in the liver (Fig. 4) furthermore resembled previously observed transcriptional changes upon phagocytosis^13^,^31^. Stress responsive genes that were upregulated following phagocytosis by PMNs, e.g. *SOD5*, *YHB1*, *GPX2*, *ASR1,* and *CAT1*, were also significantly upregulated by *C. albicans* cells in the liver (Suppl. data 8). In the kidney, in contrast, *SOD5* and *SSB1* were upregulated while other core stress response genes showed decreased expression (e.g. *HSP70*, *HSP90* and *CCP1*; Suppl. data 8). All of the stress-responsive genes with increased expression in the kidney 24 h p.i. are also known to be induced upon oxidative stress^48^, possibly indicating interaction with immigrating phagocytes at later stages of infection in the kidney. These organ-specific differential expression kinetics of stress-associated fungal genes might reflect the higher abundance of immune cells in the liver. Residential immune cells likely induce an early profound stress response in *C. albicans*, which we were able to detect in the liver 8 h p.i. It has been shown that interaction between *C. albicans* and resident macrophages also occurs in the kidney within 2 h p.i.^49^; however, this interaction does not inhibit fungal filamentation and could not be detected on day 1 p.i. The low transcription of fungal stress genes in the kidney 12 h p.i. observed by us further supports that the interaction with resident phagocytes in the kidney is only transient and that additional phagocytes need to be recruited upon infection to this organ^2^,^49^. Thus, low transcription of fungal stress genes in the kidney at the 12 h time point, followed by delayed induction (compared to liver infection) could reflect a fatal later onset of interaction with recruited immune cells in the kidney, whereas early phagocytosis by resident or rapidly recruited immune cells may contribute significantly to the control of *C. albicans* in the liver.

As discussed above, phagocytosis of *Candida* yeasts in the liver possibly exposes the fungus to an environment that prevents hyphal growth. Even though *C. albicans* filaments within macrophages *in vitro*^31^, recent findings in a zebrafish model^50^ suggest that some macrophages can inhibit filamentation *in vivo*^51^ and *C. albicans* DEGs in the zebrafish model showed a significant overlap with the transcriptome in the liver. Furthermore, phagocytosis by PMNs prevents *C. albicans* filamentation^13^,^52^. It therefore appears plausible that *C. albicans* responds to the hepatic environment by induction of a hypha-associated program while physical formation of hypha is counteracted by the activity of phagocytes. This hypothesis is supported by the observation that combined neutropenia and C5 deficiency in mice led to the development of foci of fungal invasion in liver and spleen. This was not observed in neutropenic C5-sufficient mouse strains, indicating that in the absence of neutrophils other phagocytes control *Candida* in the liver^53^. However, further studies are needed to determine the type of immune cells and exact mechanisms that prevent hypha formation and facilitate fungal clearance in the liver.

### Comparability of gene expression profiles across different studies

One very surprising result of the GSEA was that we did not observe a significant overlap with transcriptional data from *C. albicans* in the murine data published previously^15^,^16^,^21^. One explanation could be the approach used for our GSEA analysis, in which we combined data from both time points for the kidney to compare this data set to the DEGs in the liver. This initial comparison, designed to detect inter-organ differences, might partially explain why no significant enrichment was observed with other *in vivo* transcription data sets from individual organs referenced to pre-infection samples.

To analyse the comparability of our and published data sets in more detail, we performed a direct comparison (Suppl. Data 9), revealing a 31–44% overlap of DEGs identified in the different studies, with 73–90% of the overlapping DEGs displayed similar trends in expression (up- or downregulated, respectively). While this overlap may appear to be low, it should be interpreted considering that technical differences between the different studies influence the fold-change gene expression and thereby the DEGs identified depending on the cut off. For example, the NanoString technology used by Xu *et al*.^15^ is highly sensitive and might thus detect low-expression DEGs missed in our study, but is also limited to the set of genes included in the methodological set up. Similarly, RNASeq after specific enrichment procedures, as performed by Amorim-Vaz *et al*.^16^, is likely to be superior in sensitivity compared to microarrays. Amorim-Vaz *et al*. furthermore analysed time points different to our study, which will affect the direct comparison. Further technical differences hampering a meaningful comparison are the different control conditions chosen for *C. albicans in vitro* and the fungal strain used^19^.

While all these factors influence the DEGs identified in the different studies, it should be highlighted that distinct pathways (generally identified by GO analysis) were found to be induced in the kidney in all studies. This includes the upregulation of iron acquisition systems, filamentation and cell wall modification as well as specific virulence factors. This demonstrates that these pathways can be robustly identified in different settings and by different technologies and thus underlines their importance for the infection process. It also shows that pathway analyses (via GO-analysis or other bioinformatical approaches) are able to produce more robust and often more informative results than individual analysis of selected genes, for which expression levels might be strongly influenced by the chosen method.

In summary, our study clearly shows that at the transcriptional level both host responses and fungal adaptation during disseminated candidiasis are organ-specific. In the kidney, late onset of innate defence mechanisms, likely due to the comparatively low number of resident immune cells and slow recruitment of additional effector cells, facilitates fungal proliferation accompanied by filament formation, upregulation of iron acquisition mechanisms, and metabolic adaptation. Failure to control fungal growth likely drives the observed exacerbated induction of proinflammatory responses, thereby contributing to immunopathology. In contrast, innate immune factors are quickly upregulated in the liver and the fungal response indicates possible phagocytosis that might help to explain the noteworthy lack of filamentation in this organ. The distinct environments in the different organs likely drive the observed differential expression of cell wall and cell surface modifying enzymes. This in turn may lead to structural alterations that can affect interaction with immune cells and thus possibly contribute to the specific course of infections in different organs.

## Material and Methods

### Ethics statement

All animal experiments were performed in accordance with the German animal protection law and were approved by the responsible Federal State authority (Thüringer Landesamt für Lebensmittelsicherheit und Verbraucherschutz) ethics committee (beratende Komission nach §15 Abs. 1 Tierschutzgesetz; permit no. 03–009/13). The animals were cared for in accordance with the European Convention for the Protection of Vertebrate Animals Used for Experimental and Other Scientific Purposes.

### Strains and culture condition

*Candida albicans* SC5314 and the GFP expressing strain M137 (Fradin *et al*., 2005) were maintained as glycerol stocks at −80 °C. For experiments, single colonies obtained from YPD agar (1% w/v peptone, 1% w/v yeast extract, 2% w/v glucose, 2% w/v agar) were grown overnight in liquid YPD (without agar) at 30 °C or in RPMI 1640 at 37 °C in a shaking incubator.

### Murine infection model

The infection was performed as described previously^54^. Briefly, SC5314 cells from liquid YPD overnight cultures were washed twice with ice-cold PBS and adjusted to the desired concentration. The infection dose was confirmed by plating. On Day 0, three mice per time point were infected via the lateral tail vein with *C. albicans* ranging from 2.5 × 10^4^ to 6.25 × 10^6^ cfu/g body weight. Female BALB/c mice of 10–12 weeks were used for infection. The remaining infection solution was centrifuged and the pellet was snap frozen in liquid nitrogen and stored at −80 °C for later RNA extraction.

After infection, the health status of the mice was examined twice a day by a veterinarian, and surface temperature and body weight were recorded daily. Mice were humanely sacrificed 8 h, 12 h, 24 h, and 72 h post infection. Immediately after euthanasia, kidneys, spleen, and liver were removed aseptically, rinsed with sterile PBS and snap frozen in liquid nitrogen. PBS mock-infected animals served as controls.

### RNA isolation

We designed an RNA isolation protocol for isolation of both fungal and murine RNA from the same sample based on step-wise isolation of host and fungal RNA (Suppl. Fig. 5). First, organs were aseptically homogenized in RLT buffer (Qiagen) with 1% β-Mercaptoethanol (β-ME) on ice water using an Ika T10 basic UltraTurrax homogenizer. Then, homogenates were centrifuged at 3,000 g at 4 °C for 3 min. The supernatant was used for mouse tissue RNA isolation with the RNeasy Mini Kit (Qiagen) as described by the manufacturer. The remaining pellets were vortexed on a mini-beadbeater (Precellys) for 5 sec at 5000 m/s in 1 ml RLT buffer (Qiagen) with 1% β-ME. After centrifugation at 4 °C, fungal RNA was isolated from the remaining pellets as previously described^24^. The RNA quality was determined using a Bioanalyzer (Agilent Inc.), the quantity was measured with a Nanodrop ND1000 (Peqlab).

### Gene expression profiling

Genome-wide gene expression of *C. albicans* was analysed with *C. albicans*-specific microarrays (ClinEuroDiag). The red channel represents hybridization with Cy5-labeled *C. albicans* RNA from experimental mouse sepsis while the green channel always shows hybridization with Cy3-labeled RNA from *C. albicans* yeast cells growing logarithmically in standard YPD medium at 37 °C with shaking (common control). *C. albicans* RNA labelling, microarray hybridization, and scanning were performed as previously described^24^.

Genome-wide gene expression profiling of mouse samples was performed using the MouseRef-8 Expression and MouseWG6 v2.0 BeadChips (Illumina) according to the manufacturer’s instruction. From the RNA isolation, 200 ng total RNA with a Bioanalyzer RIN greater than seven were used for amplification prior to chip hybridization. Samples were analysed using the iScan platform (Illumina) measuring the variation of expression rate of > 42,000 transcripts. All microarray data are MIAME compliant and raw data have been deposited at GEO (GSE83682). The gene expression of liver, spleen, and kidney tissue 8 h, 12 h, 24 h, and 72 h after intravenous infection was compared to control samples of mock infected mice 24 h after PBS injection.

### Expression data analyses

All analyses were performed in R using packages provided by Bioconductor 2.26^55^. *C. albicans* two-colour microarray data were pre-processed using the limma package^56^. “Printtiplowess” normalisation was used on each array separately to correct for spatial effects or cross-hybridization. Array spots corresponding to the same gene were summarized using the duplicated correlation function of limma. Normalization of the arrays was performed using between-array quantile normalization. Log2 fold-changes (log2-FC) were calculated between *in vivo* samples and the common reference using limma. Genes with Benjamini-Yekutieli corrected p-value < 0.05 were considered as differentially expressed.

Mouse data was annotated using appropriate Illumina manifest files. For each platform raw data were pre-processed independently using the lumi package^57^ for R, including background correction (bgAdjust) and subsequent data normalization using variance stabilization transformation and quantile normalization. Detection calls for each probe were performed at default parameters settings and probes detected as absent in all samples were removed. Subsequently, the illuminaMousev2.db package for R was used for re-annotation and probe filtering. We retained only probes with a quality grade of “good” or “perfect”. The normalized and pre-processed data set for the two different microarray platforms was integrated into a combined data set by probe ID matching and subsequent between array loess normalization (affy package 1.44; ref. 58) with the “span” parameter set to 0.1. Quality of the integration procedure was verified by analysis of the expression values of known housekeeping genes and identified stable reference genes before and after loess normalisation. Differential expression was assessed using limma with a Benjamini-Yekutieli corrected p-value below 0.05 and an absolute log2-FC of 0.5 for each organ and time-point. To compare expression values of the control samples between organs we included the within-donor correlation for samples derived from the same animal estimated using the duplicate Correlation function provided by limma.

GO-term analysis of *C. albicans* expression data was performed using FungiFun2^59^. The results contain GO-categories with Fisher’s exact test determined, Benjamini-Hochberg corrected p-values below 0.05.

### Cluster analysis

Clustering of differentially expressed genes was performed using the clValid package^60^ for R, allowing direct comparison of the results from various clustering methods. For our analysis, we compared the results of 5 clustering algorithms (*Hierarchical clustering*, *k-means*, *SOTA*^61^, *Diana* and *Clara*^62^) for 3 to 15 clusters. The resulting scores of each validation measure were scaled between 0 and 1 and transformed such that a score of 1 represents the best result. Subsequently, all scores were combined using the average of the mean internal score and the mean stability score. Finally, we selected the clustering method and the number of clusters according to the maximal overall score. Subsequent gene ontology enrichment analyses for the genes of each cluster were performed using the GOstats package^63^ excluding all terms with less than 10 or more than 500 genes.

### Gene Set Enrichment Analysis (GSEA)

For gene set enrichment analysis (GSEA) a gene list was produced from (i) the combined DEGs of *C. albicans* in kidney 12 h and 24 h and (ii) liver 8 h p.i., which were imported as “phenotypes” to be compared into the GSEA software v2.2.0 (Broad Institute)^64^. Analysis parameters were as follows: norm, meandiv; scoring_scheme, weighted; Metric:Diff_of_Classes; set_min, 15; nperm, 1000; set_max, 5000. Gene sets with an FDR < 25% were considered as significant enriched. This set was used for a gene set enrichment analysis (GSEA) based on the recently published list of regulated genes compiled from the literature^16^ expanded with another recently performed *in vivo* transcriptional profile of *C. albicans*^15^ and the gene list “GSEA_Nantel_2012” kindly supplied to CGD by Andre Nantel (www.candidagenome.org/download/community/GSEA_Nantel_2012/).

### Inference of pathogen-host gene-regulatory network

The ExTILAR algorithm^33^ was used for the inference of an inter-species pathogen-host gene-regulatory network (GRN) based on prior knowledge about transcription factor binding sites (TFBSs) and about genes known to affect the activity of transcription factors. oPOSSUM 3.0^65^ was used to detect over-represented TFBSs for each cluster. For clusters formed by the genes of *M. musculus* we restricted the promoter region of the target genes between 2,000 base pairs (bp) upstream and 0 bp downstream of the transcription start site. TFs corresponding to TFBSs with a Z-score of 10 or more and a Fisher-score of 7 or more (default values) were considered as potential regulators of the respective cluster. For *C. albicans*, the *S. cerevisiae* tool required the mapping of ORF-IDs to gene symbols of the *Saccharomyces* Genome Database (SGD). oPOSSUM was then used at default values restricting the number of background genes to 5,000 randomly selected. TFs corresponding to TFBSs with a Z-score of 10 or more and a Fisher score of 5 or more were considered as potential regulators of the respective cluster.

Based on the temporal mean cluster expression profile and the respective standard deviation (SD) as well as the TF-to-gene information obtained from oPOSSUM we inferred 10,000 networks. For each inference, we sampled expression data from a normal distribution using the mean and SD of the respective cluster profile/time point. ExTILAR was applied at default parameters including stepwise forward selection for the optimization of the structure-template. The final stable consensus network was derived by selection of edges between clusters that were inferred in more than 50 percent of all networks. Using the stable consensus network structure as a template we applied an ordinary least squares approach for parameter estimation of the final network model.

### Real time qPCR

For microarray validation and expression analysis of core filamentation response genes, qRT-PCR was performed using the my-Budget 5 × EvaGreen QPCR Mix II (Bio&Sell) in a C1000TM Thermal Cycler (BioRad) according to manufacture recommendations. Gene specific primers are listed in Suppl. Table 2. Relative gene expression levels were determined by the 2^ΔΔCt^ method with *ACT1* and *EFB1* as housekeeping genes. RNA isolated from the *C. albicans* infection solution served as reference. Murine RNA from mock-infected mice served as negative control.

## Additional Information

**How to cite this article**: Hebecker, B. *et al*. Dual-species transcriptional profiling during systemic candidiasis reveals organ-specific host-pathogen interactions. *Sci. Rep.* **6**, 36055; doi: 10.1038/srep36055 (2016).

**Publisher’s note**: Springer Nature remains neutral with regard to jurisdictional claims in published maps and institutional affiliations.

## Supplementary Material

Supplementary Information

Supplementary Dataset 2

Supplementary Dataset 3

Supplementary Dataset 4

Supplementary Dataset 5

Supplementary Dataset 6

Supplementary Dataset 7

Supplementary Dataset 8

Supplementary Dataset 9

Supplementary Dataset 10

## Figures and Tables

**Table 1:** List of fungal genes significantly upregulated by *C. albicans* in the liver 8 h p.i.

orf	Name	Liver 8 h	Kidney 12 h	Kidney 24 h
orf19.3374	*ECE1*	10.535	4.930	7.899
orf19.2060	*SOD5*	8.370	4.242	4.074
orf19.5741	*ALS1*	6.613	2.065	2.277
orf19.6037	*ASM3*	5.801	2.351	2.414
orf19.1321	*HWP1*	5.765	4.585	3.079
orf19.2942	*DIP5*	4.992	1.723	1.530
orf19.6993	*GAP2*	3.758	−1.404	−1.459
orf19.4975	*HYR1*	3.708	1.906	2.603
orf19.2355	*ALS3*	3.657	2.471	2.412
orf19.85	*GPX2*	3.203	1.483	1.783
orf19.5760	*IHD1*	3.180	1.850	1.870
orf19.6367	*SSB1*	2.925	1.975	1.637
orf19.711		2.802	3.277	1.592
orf19.7469	*ARG1*	2.762	1.222	1.276
orf19.7114	*CSA1*	2.669	8.687	9.024
orf19.5916		2.599	1.439	−1.435
orf19.4456	*GAP4*	2.314	1.896	2.056
orf19.7084	*DFI1*	2.286	1.371	1.479
orf19.6844	*ICL1*	2.268	1.562	1.637
orf19.2608	*ADH5*	2.075	−2.394	−1.873
orf19.6837	*FMA1*	2.047	1.376	−1.635
orf19.3384		2.040	1.273	1.526
orf19.3829	*PHR1*	2.014	1.542	1.604
orf19.5170	*ENA21*	1.931	1.478	2.167

Values indicate absolute log2-FC. Note, that *ADH5* and *GAP2* were significantly upregulated in the liver 8 h p.i., but downregulated in the kidney at both time points.

**Table 2:** Gene expression of the core filamentation response determined by qPCR and microarray.

Gene name	Liver 8 h		Liver 12 h	Kidney 12 h		Liver 24 h	Kidney 24 h	
	qPCR	Microarray	qPCR	qPCR	Microarray	qPCR	qPCR	Microarray
*ALS3*	80.6	1.9	84.8	89.2	*1.1*	139.3	62.4	*1.2*
*DCK1*	1.8	*1.6*	1.3	0.9	*1.2*	1.2	0.8	1.5
*ECE1*	793.5	10.5	629.3	3061.2	4.9	1817.4	3654.9	7.9
*HGT2*	150.3	5.3	288.5	71.2	*1.4*	194.6	127.4	*1.5*
*HWP1*	292.2	5.8	128.2	201.4	4.6	81.6	172.0	3.1
*IHD1*	17.2	3.2	9.2	10.1	1.9	9.2	13.9	1.9
*RBT1*	1.1	*1.0*	9.6	7	*0.9*	2.0	7.2	*0.9*
orf19.2457	3.7	1.8	5.1	2.7	1.4	4.3	3.3	1.4

Values in italic were not detected as significant differentially expressed by microarray analysis. Overall, gene expression quantified by qPCR (fold change determined by 2^ΔΔCt^ method) and microarray (absolute log2-FC), respectively, show the same trend in regulation.
