# Corrigendum: Dual-species transcriptional profiling during systemic candidiasis reveals organ-specific host-pathogen interactions

**DOI:** 10.1038/srep39423

**Published:** 2016-12-19

**Authors:** Betty Hebecker, Sebastian Vlaic, Theresia Conrad, Michael Bauer, Sascha Brunke, Mario Kapitan, Jörg Linde, Bernhard Hube, Ilse D. Jacobsen

Scientific Reports 6: Article number: 3605510.1038/srep36055; published online: 11 03 2016; updated: 12 19 2016

This Article contains an error in the legend of Table 1.

“Values indicate absolute log2-FC.”

should read:

“Values indicate absolute fold-change.”

