# Supplementary material for: Dual-species transcriptional profiling during systemic candidiasis reveals organ-specific host-pathogen interactions
Source: Sci Rep. 2016 Nov 3;6:36055. doi: 10.1038/srep36055 (PMC5093689; doi:10.1038/srep36055)
Supplement: Supplementary Information [file srep36055-s1.pdf]

Supplementary information

Dual-species transcriptional profiling during systemic candidiasis reveals organ-specific host-pathogen interactions.

Betty Hebecker, Sebastian Vlaic, Theresia Conrad, Michael Bauer, Sascha Brunke, Mario Kapitan, Jörg Linde, Bernhard Hube, Ilse D. Jacobsen

---

Supplementary Figures

Supplementary Figure 1. Differentially regulated genes in liver, kidney and spleen after systemic *C. albicans* infection. A) Venn diagram of differentially regulated genes (DEGs) and corresponding significantly enriched GO terms according to cluster analysis. GO terms (condensed using REVIGO: http://revigo.irb.hr/) enriched in positively regulated genes are shown in a red box, negatively regulated genes in a green box. Green filled boxes show GO terms enriched in liver, spleen and kidney. Overlap all organs: Genes differentially regulated in all organs. **B)** Clustering of differentially expressed genes over time and organ. Nine cluster represent the changes in gene expression after systemic *C. albicans* infection. For each cluster, scaled expression profiles of the assigned genes are shown in gray. Cluster mean expression profile (solid lines) and standard deviation (dashed line) are shown for each cluster and organ respectively. Cluster designation and the number of genes in each cluster are shown above each panel. Mean log2-expression ratios for genes that are differentially expressed in at least one time point ($p < 0.05$ with more than 0.5 change of transcript abundance between treatment and control) were used for K-means clustering.

A complete list of all significant GO terms within the distinct clusters is provided as Supplementary data 2.

Supplementary Figure 1

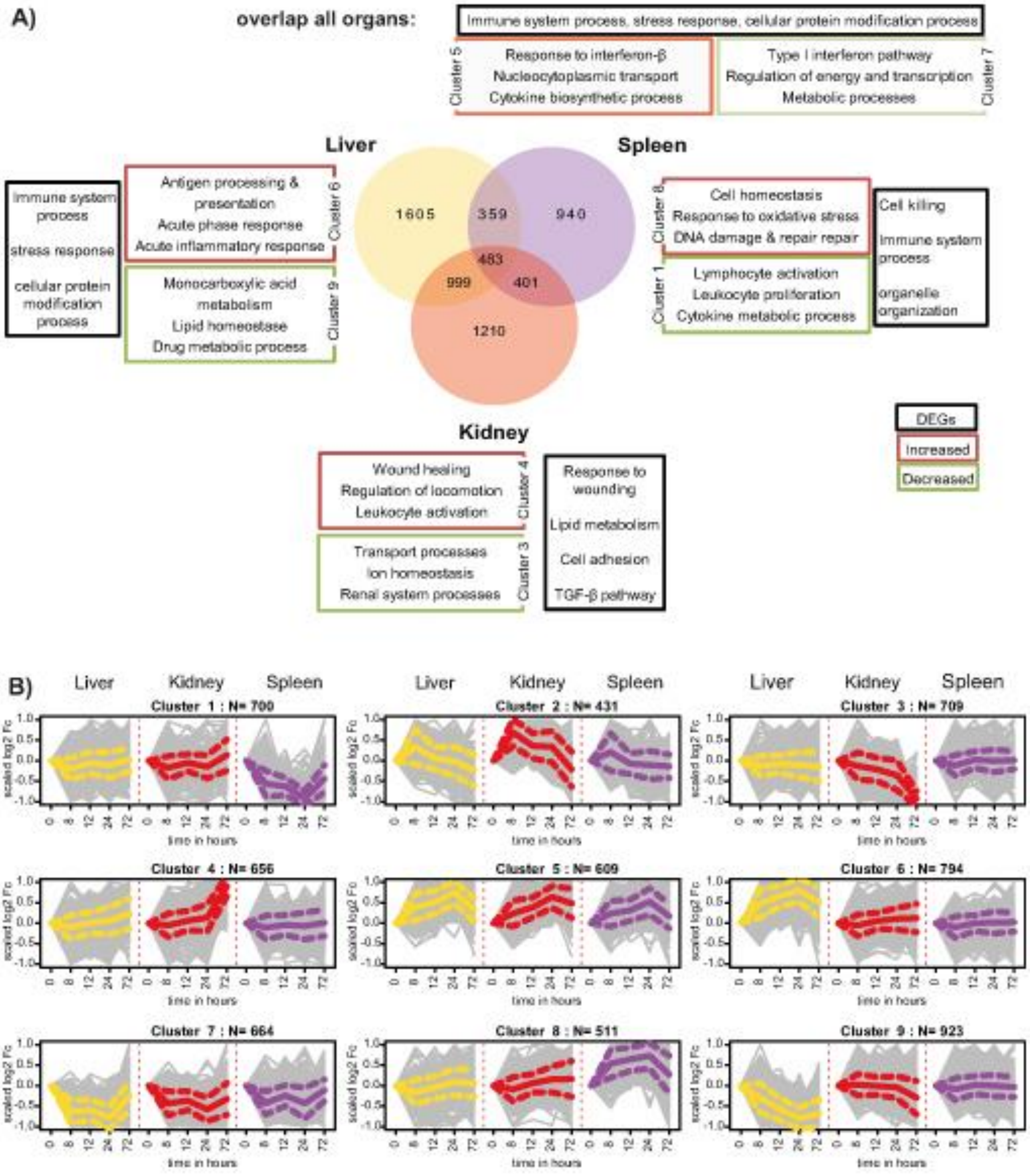

Supplementary Figure 2: Dendrogram of *C. albicans* DEGs profiles and clustering of expression profiles for the inference network. A) Dendrogram of the similarity of DEG profiles in liver and kidney at different time points. **B)** and **C)** Cluster used for the inference network. Shown are mean expression profile (solid lines) and standard deviation (dashed line) for each cluster and organ, respectively. The clustering analysis contains all genes showing an increased or decreased expression at at least one time point. B) *C. albicans* DEGs; C) murine DEGs.

A complete list of all significant GO terms within the distinct clusters is provided as Supplementary data 4.

A)

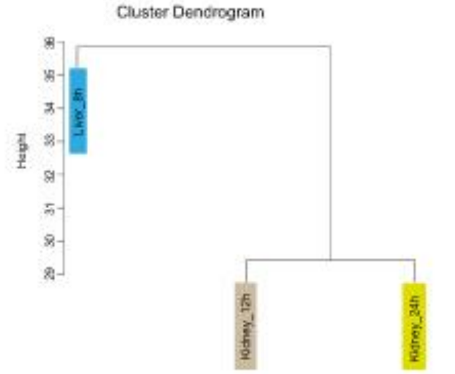

Supplementary Figure 2

B)

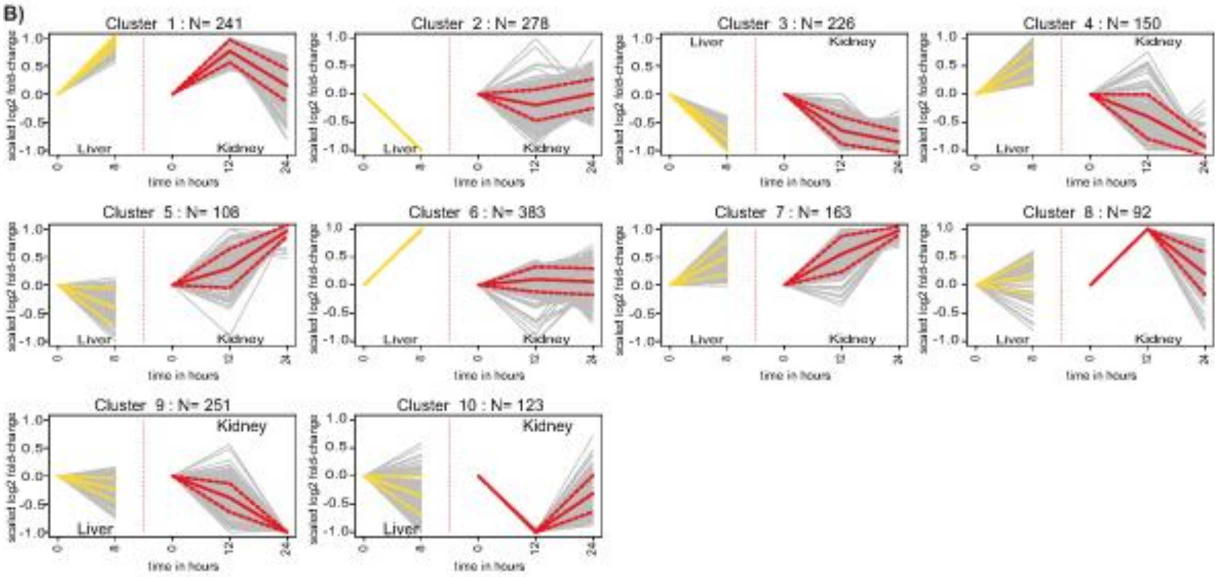

C)

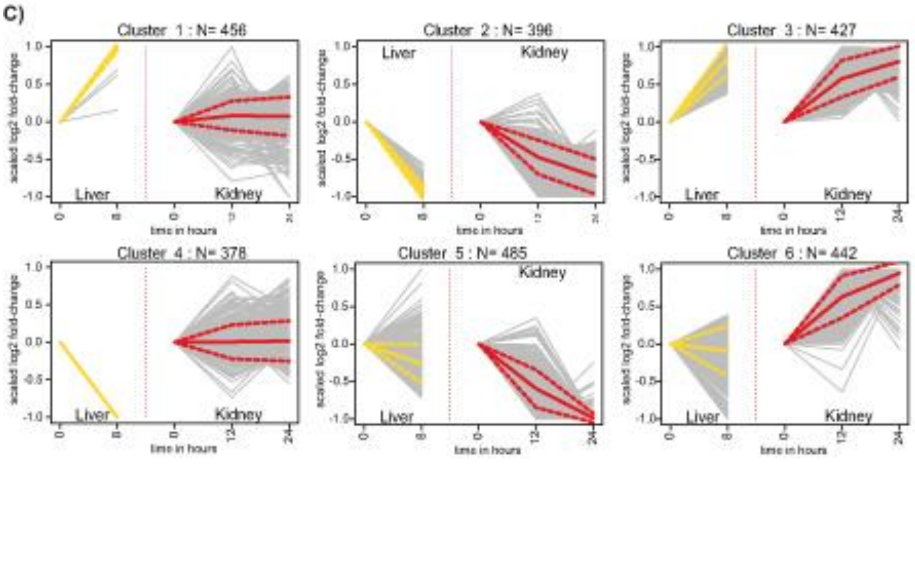

Supplementary Figure 3: qPCR analysis of the core filamentation response genes. In addition to the RNA samples used for microarray analysis, fungal RNA from infected liver 12h and 24h p.i. were included in the analysis. Fold change expression in *in vivo* samples compared to the CR (log-phase SC5314 grown in YPD), normalized to ACT1, is shown. n=3

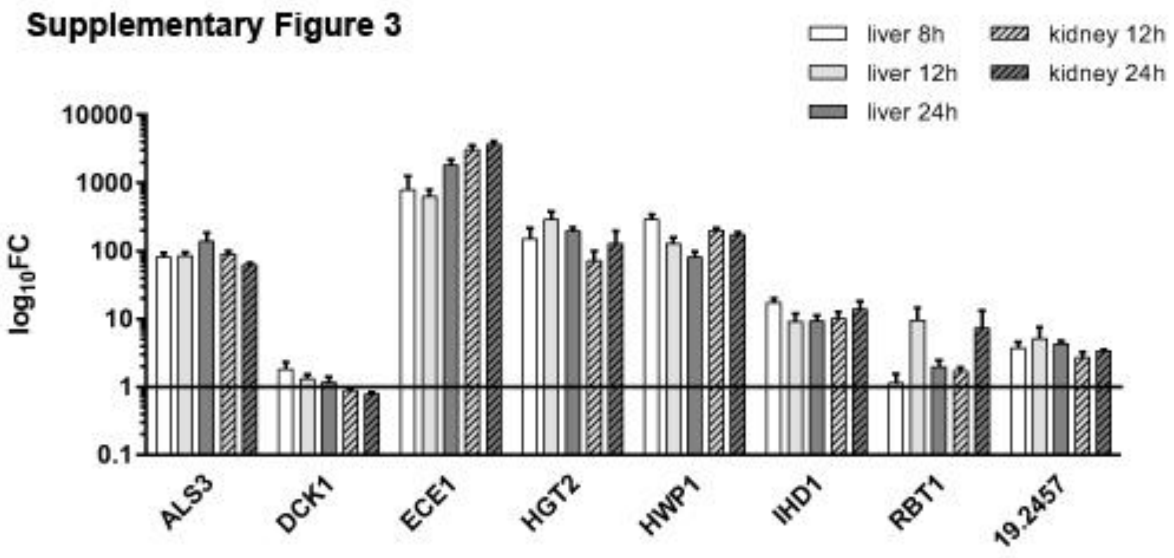

Supplementary Figure 4. Interaction of *C. albicans* yeast cells under hypha-inducing conditions with human primary macrophages. A) Morphology of *C. albicans* after incubation in RPMI. Over a time period of 30 min, no germ tube formation was detectable. First germ tube formation was detectable 45 min after *C. albicans* cells were transferred to RPMI, however, more than 90% of the cells grew in the yeast form. B) Representative images of human MDMs co-incubated with *C. albicans* yeast cells at an MOI of 5 for 30 min. Expression of filamentation genes was not induced (top, 0 min RPMI) or induced for 30 min in RPMI (bottom). After differential staining the phagocytosed cells appear in blue, whereas non-phagocytosed cells appear in pink. C) Percentages of *C. albicans* cells phagocytosed by human MDM. Percentage phagocytosis was determined by analysing the uptake of *C. albicans* cells compared to all fungal cells detected on an area of around 5 mm² after co-incubation for 30 min at an MOI of 5. Human MDMs were isolated from blood of six different healthy donors; the experiment was performed in duplicates for each donor. * $p < 0.05$, t-test, each time point compared to the previous time point. D) Cytokine release of human primary macrophages 24h after co-incubation with thimerosal-killed *C. albicans* SC5314 and CAI4-pACT1-GFP (M137), n = 4. The amount of cytokine release was measured by ELISA and plotted as percentage of cytokine release of zero minutes of hyphal induction (100%). Cytokine concentrations of MDMs coincubated for 24h with yeast were in the following ranges (mean in pg/ml): TNF-a, 978.8 (0 min) 341.4 (15 min) 361.1 (30 min) 1053 (45 min); IL-8, 1877 (0min) 508.4 (15 min) 596.3 (30 min) 1331 (45 min).

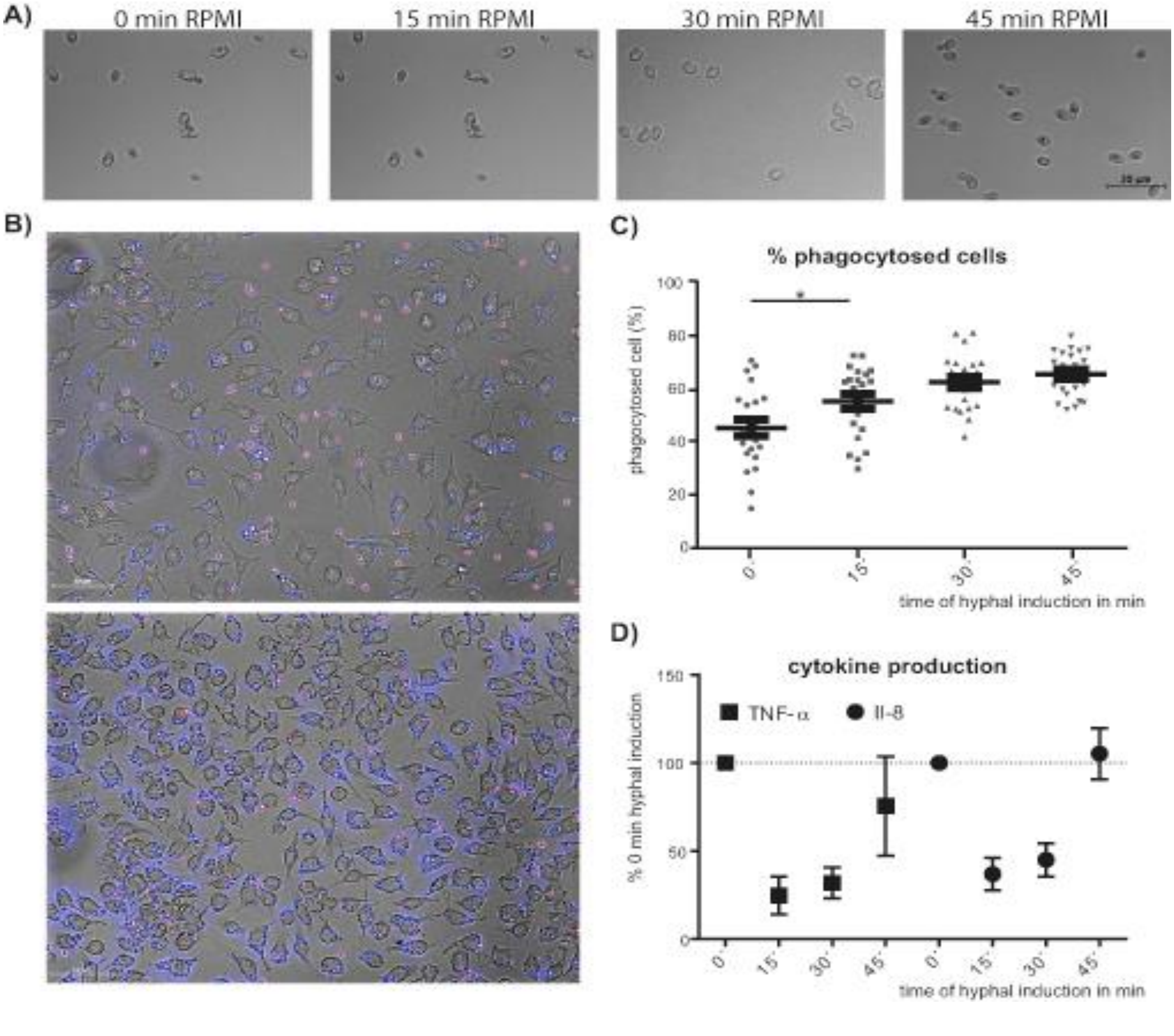

Associated Material and Methods for Suppl. Fig. 4:

Macrophage culture

Human peripheral blood mononuclear cells (PBMC) were isolated by Histopaque-1077 (Sigma-Aldrich) density centrifugation from buffy coats donated by healthy volunteers as described previously[60]. Macrophage infection experiments were performed in RPMI 1640 without serum and without M-CSF. All experiments were performed with cells isolated from at least three different donors.

Phagocytosis assay

For phagocytosis experiments, the wild-type *C. albicans* strain SC5314 and the GFP-expressing strain M137 were grown in liquid YPD. After harvesting, the cells were transferred either into liquid YPD at 30°C (0 min hypha induction, control) or RPMI 1640 at 37°C in a shaking incubator. Cells were then harvested by centrifugation after 15 min, 30 min, or 45 min. MDMs seeded in 24-well plates with coverslips were infected with these cells induced for hyphal gene expression for 0 min, 15 min, 30 min, and 45 min in RPMI at an MOI of 2 in duplicates. After 30 min of co-incubation the MDMs were extensively rinsed with PBS to remove non-adherent cells. Cells were fixed with 4% paraformaldehyde for 10 min at 37˚C, and stained for 30 min at 37˚C with 25 mg/ml Alexa Fluor 647-conjugated Con A (Molecular Probes) to visualize non-phagocytosed yeast cells. After permeabilization all *C. albicans* cells were stained with calcofluor white (Fluorescent brightener 28, Sigma) for 15 min and mounted cell side down in ProLong Gold antifade reagent (Molecular Probes). Uptake by MDMs was assessed by fluorescence microscopy using an Axio Observer with the Zen 2012 software (Zeiss Inc.) Using the MosaiX tile scan images from an area of around 5 mm^2 per cover slip were taken and analysed by ImageJ. Additionally, the phagocytosis rate was counted manually for two donors per experiment on an area of 0.33 mm^2 per cover slip in duplicates. The experiments were performed in duplicate with MDMs isolated from six different donors.

Cytokine production

MDMs were infected with thimerosal-killed *C. albicans* yeast cells in 24-well plates at an MOI of 10. Lipopolysaccharide (LPS) (Sigma-Aldrich) was used as a control and applied at a concentration of 1µg/ml. To prepare thimerosal-killed cells, the strains were grown overnight in YPD, resuspended at a concentration of 10^8 cells in 25 ml pre-warmed RPMI or YPD and incubated for 15, 30 or 45 min at 37°C in RPMI and 45 min at 30°C in YPD (0 min of hypha induction, control) in a shaking incubator followed by centrifugation. Pellets were resuspended in water with 0.5 mM thimerosal (Sigma-Aldrich) and incubated for one hour at 37°C in a shaking incubator. After extensive washing with water, the cells were frozen overnight at -20°C in PBS. After thawing, the cells were adjusted to a concentration of 2×10^7 cells/ml and used for MDM infection. Aliquots were plated on YPD to control the killing efficiency. After 24h of co-incubation, samples of surrounding medium were collected and centrifuged (10 min, 1,000 x g). The amount of TNF-α and Il-8 secreted by MDMs was determined by enzyme-linked immunosorbent assay (ELISA) according to the protocol of the manufacturer (eBioscience). All experiments were performed in duplicates and normalized to the control sample (45 min growth in YPD at 30°C).

Supplementary Figure 5: Overview of the sample preparation procedure. *C. albicans* was intravenously injected into mice. After 8h, 12h, 24h, and 72h the mice were humanely sacrificed and liver, kidney and spleen aseptically removed. After RNA isolation and confirmation of sufficient RNA quality, *C. albicans* RNA was Cyanin (Cy) labeled to hybridize microarrays. RNA isolated from mouse organs were used for hybridizing mouse BeadChips.

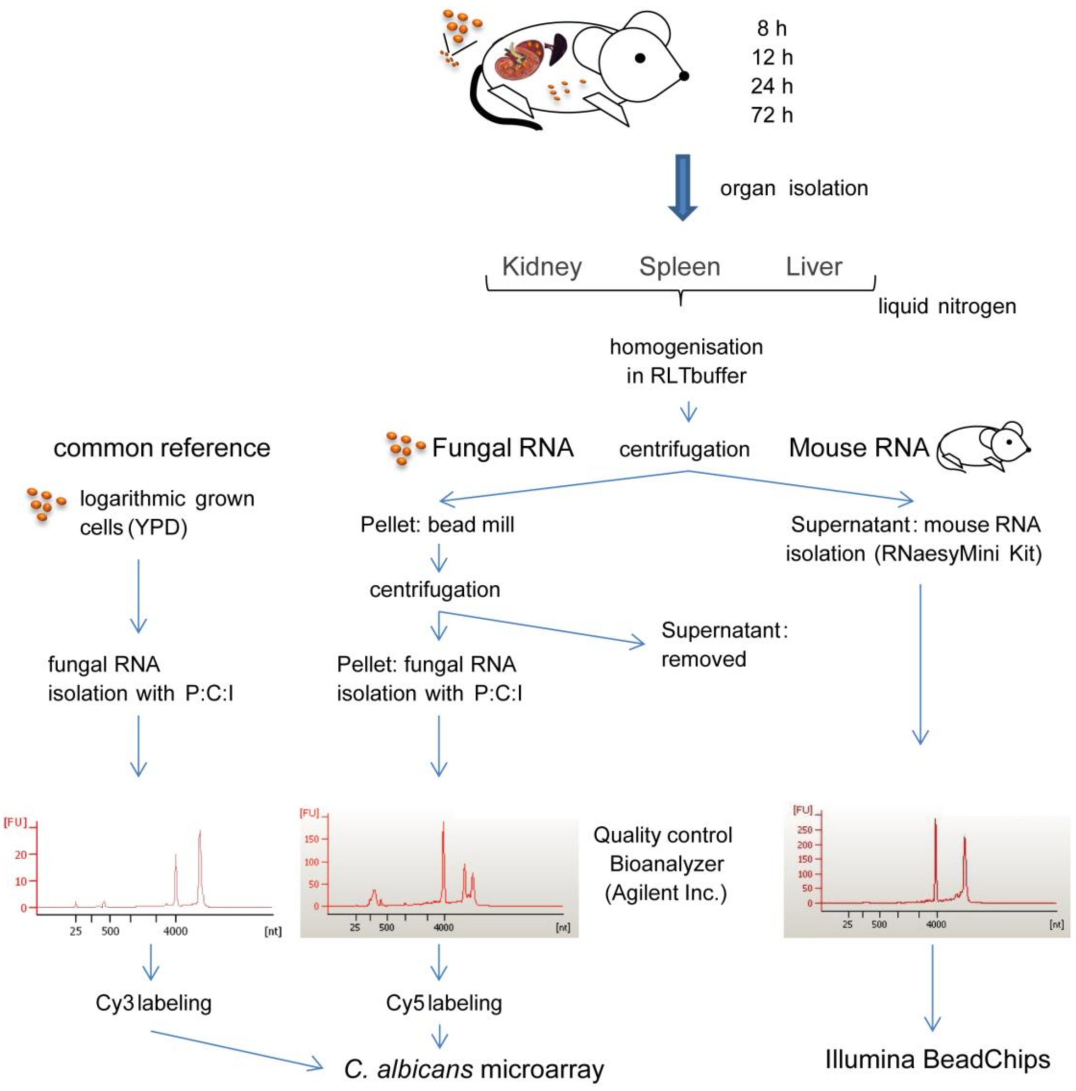

Supplementary Tables

		Liver 8h		Kidney 12h		Kidney 24h	
name	orf	qPCR	Microarray	qPCR	Microarray	qPCR	Microarray
NRG1	orf19.7150	n.a.	not det.	0.05	-1.34	n.a.	not det.
CAT1	orf19.6229	4.26	2.49	1.41	0.093	0.19	-0.37
HYR1	orf19.4975	132.21	3.71	172.87	1.91	8.26	2.6
SOD5	orf19.2060	4865.13	8.37	1426.58	4.24	2624.02	4.07
PRA1	orf19.3111	n.a.	not det.	717.79	1.76	n.a.	not det.
ZRT1	orf19.3112	n.a.	not det.	741.75	1.77	n.a.	not det.
ZRT2	orf19.1585	n.a.	not det.	1.12	0.31	n.a.	not det.

Supplementary Table 1: qRT-PCR confirmation of microarray results for selected *C. albicans* genes. *NRG1* was downregulated in kidney 12h p.i., *HYR1*, *SOD5* were upregulated at all analysed samples. *CAT1* was organ specific regulated (upregulated in liver, downregulated in kidney 24h p.i.). *PRA1* and *ZRT1* were upregulated in kidney 12h p.i.. *ZRT2* was not differentially expressed. n.a. = not analyzed; not det. = not detected by microarray analysis; grey values = not significantly differentially expressed

Supplementary Table 2: Primers and conditions for qPCR

target gene	sequence in 5'-3' direction
ALS3	ATGGTCCTTATGAATCACCATCTA
	TAGCAGTTGTAGTTGTAGATGGAG
DCK1	TCGATGAAACTGTCACATCA
	TGATGCTCTACCTGATCTAGTG
ECE1	ATCGAAAATGCCAAGAGAG
	AGCATTTTCAATACCGACAG
HGT2	TGCTAATTGGATTTTGAATTTCGCTA
	TGATGTTCAACTTCCATCTTTCTTG
HWP1	ATCAGCTCCTGCCACTGAAC
	TGAGTGGAACTGATTCTAATGTAGTTG
IHD1	ATGGAACCAGCAGCAGATC
	AGTAGCAGCAAAACCACCAG
RBT1	CTACTCCAGTTGCACCAGTTG
	CAAGACCAATAATAGCAGCACC
orf19.2457	AGACTCGCCAGAATTGGCTCA
	TGCCATGGGGATCAGATTCAG
ACT1	TCAGACCAGCTGATTTAGGTTTG
	GTGAACAATGGATGGACCAG
EFB1	AGTCATTGAACGAATTCTTGGCTG
	TCTTCATCAACTTCATCATCAGAACC

HYR1	ACTGAATCTAAACCTGGTTTC
	TACCACCAGTAACAATAGATG
NRG1	CACCTCACTTGCAACCCC
	GCCCTGGAGATGGTCTGA
CAT1	ACTCCAGTGTTTTTCATTAGAG
	AGAGTAACCATTCATTTCTCTG
ZRT1	ACGCTGTGACTTGTGAAAGAG
	CACATTAATTGCGCATGAGTC
ZRT2	CAGACACAGATATACTCCATGGATT
	AATTCGTGTGCCATCAATTC
PRA1	TGAGGTCGTTGGTCATTTTG
	ACCGGAGCATAGTTGGGATA
SOD5	GGAGCAGTAGAAGCCATACTA
	ATGGGCGAGTCCTACAAAACT

Program
1 95.0 °C for 15:00 min
2 95.0 °C for 0:15 min
3 59.0 °C for 0:20 min
4 72.0 °C for 0:20 min + Plate Read
5 GOTO 2, 39 more times
6 95.0 °C for 0:15 min
7 Melt Curve 65.0 °C to 95.0 °C, increment 0.5 °C, 0:15 min + Plate Read

Supplementary Data

Supplementary Data 1: List of DEGs mouse

Supplementary Data 2: GO terms cluster mouse

Supplementary Data 3: List of DEGs *Candida albicans*

Supplementary Data 4: GO terms *Candida albicans*

Supplementary Data 5: GO terms mouse cluster inference model

Supplementary Data 6: GO terms *Candida* cluster inference model

Supplementary Data 7: GSEA *Candida albicans*

Supplementary Data 8: core stress responses *Candida albicans*

Supplementary Data 9: Comparison with published data sets

Details for Supplementary Data 9:

The published data was processed for the comparison as follows:

For comparison of murine transcription data, preprocessed data published by MacCallum, 2009, FEMS Yeast Research, was downloaded from ArrayExpress (E-MEXP-1458) and log2 transformed. Affymetrix ProbeSetIds were mapped onto EntrezGeneIds, multiple ProbeSetIds representing the same EntrezGene-Id were averaged. For the virulent strain NGY152 samples were compared to the mean of the control samples. Genes with an absolute fold-change greater 1.5 (log2 (1.5)) were denoted as differentially expressed. In total 396 DEGs were identified in both data sets (MacCallum and Hebecker *et al.*, respectively). 357 of these 396 DEGs showed identical direction of regulation, corresponding to an excellent agreement of 90% between the shared DEGs. To facilitate comparison of our data published by that of Xu *et al.* 2015, PLOS Genetics, fold-changes from wild-type time courses were obtained from supplementary file 3 of Xu *et al.* and log2 transformed. Xu *et al.* reported 34 DEGs for the 12h comparison and 39 DEGs for the 24h comparison. Of these DEGs, we identified an overlap of 2 DEGs for the 12h comparison and 7 DEGs for the 24h comparison. Direction of regulation was identical for all shared DEGs, corresponding to an agreement of 100%.

To compare the transcriptomes of *C. albicans*, preprocessed data was obtained from supplementary file S1 and S4 of Xu *et al.* and log2 transformed. Thereby, 190 DEGs for the 12h comparison and 173 DEGs for the 24h comparison were reported. The comparison with our data showed an overlap of 59 DEGs for 12h and 58 DEGs for 24h. 52 out of these 59 (88%) and 47 out of the 58 (81%) shared DEGs showed an identical direction of regulation. Furthermore, log2-fold-changes of expressed genes were downloaded from supplementary file sd1 of Amorim-Vaz *et al.*. Amorim-Vaz *et al.* detected 1468 DEGs for the 16h comparison and 2117 DEGs for the 48h comparison. We identified an overlap of 265 DEGs by comparing the 16h DEGs dataset of Amorim-Vaz *et al.* with our DEGs for 12h, 285 shared DEGs for 16h and 24h and an overlap of 387 DEGs for the comparison of 48h and 24h. All datasets of shared DEGs showed an agreement of 73% regarding the identical direction of regulation.

Note that the data set of Andes et al. was not included in the comparison as infection was performed in leukopenic mice, thereby substantially altering the host environment.

References:

Amorim-Vaz, S. *et al.* RNA Enrichment Method for Quantitative Transcriptional Analysis of Pathogens In Vivo Applied to the Fungus *Candida albicans*. *mBio* **6,** e00942-915 (2015).

MacCallum, D. M. Massive induction of innate immune response to *Candida albicans* in the kidney in a murine intravenous challenge model. *Fems Yeast Res.* **9,** 1111–1122 (2009).

Xu, W. *et al.* Activation and alliance of regulatory pathways in *C. albicans* during mammalian infection. *PLoS Biol.* **13,** e1002076 (2015).
